# Supplementary material for: Boron-Doped Reduced Graphene Oxide with Tunable Bandgap and Enhanced Surface Plasmon Resonance
Source: Molecules. 2020 Aug 11;25(16):3646. doi: 10.3390/molecules25163646 (PMC7465222; doi:10.3390/molecules25163646)
Supplement: Supplementary file 1 [file molecules-25-03646-s001.pdf]

Supporting Material

Boron Doped Reduced Graphene Oxide with Tunable Bandgap and Enhanced Surface Plasmon Resonance

Muhammad Junaid 1,2*, M. H. Md Khir 1,*, Gunawan Witjaksono 3, Nelson Tansu 5, Mohamed Shuaib Mohamed Saheed 4, Pradeep Kumar 1, Zaka Ullah 1*, Asfand Yar 6 and Fahad Usman 6

[1] Department of Electrical and Electronic Engineering, Universiti Teknologi PETRONAS, 32610 Seri Iskandar, Perak, Malaysia.; Harisk@utp.edu.my (M.H.M.K.), Pradeep.hitesh@gmail.com (P.K.); Zaka_18000817@utp.edu.my (Z.U.)

[2] Department of Electronic Engineering, Balochistan University of Information Technology, Engineering, and Management Sciences, 87300 Quetta, Balochistan, Pakistan.

[3] BRI Institute, Jl. Harsono RM No.2, Ragunan, Passsar Minggu, Jakarta 12550, Indonesia. Gunawan.witjaksono@gmail.com (G.W.)

[4] Department of Mechanical Engineering, Universiti Teknologi PETRONAS, 32610 Seri Iskandar, Perak, Malaysia. Shuaib.saheed@utp.edu.my (S.S.)

[5] Center for Photonics and Nanoelectronics, Department of Electrical and Computer Engineering, Lehigh University, 7 Asa Drive, Bethlehem, PA 18015, USA. Tansu@lehigh.Edu (N.T.)

[6] Department of Fundamental and Applied Sciences, Universiti Teknologi PETRONAS, 32610 Seri Iskandar, Perak, Malaysia.; Asfandyarhargan@gmail.com (A.Y.); Fahad_17004459@utp.edu.my (F.U.)

* Correspondence: Muhammad_17000796@utp.edu.my (M.J.)

1- Electrical conductivity Measurement

The electrical conductivity of GO and B-rGO samples measured at room temperature using Hall-effect measurement (HMS-3000 Series) is shown in Table 1. The electrical conductivity of GO increases with the reduction of oxygen-related functional groups and an increase in sp^2 domains. The electrical conductivity of GO was found to be further improved with boron doping, and this might be dependent on the doping concertation of incorporated atoms [1]. The increment in p-type electrical conductivity corresponds to the increase in boron contents in the B-rGO samples. These results are found to be in agreement with previous studies [2,3].

Table S1. The electrical conductivity of GO and B-rGO.

S/No	Material	Conductivity (S/cm)
1	GO	0.127
2	B-rGO1	0.725
3	B-rGO2	0.767
4	B-rGO3	0.816

References

1. Khai, T. Van; Na, H.G.; Kwak, D.S.; Kwon, Y.J.; Ham, H.; Shim, K.B.; Kim, H.W. Comparison study of structural and optical properties of boron-doped and undoped graphene oxide films. *Chem. Eng. J.* **2012**, *211–212*, 369–377.

2. Umrao, S.; Gupta, T.K.; Kumar, S.; Singh, V.K.; Sultania, M.K.; Jung, J.H.; Oh, I.K.; Srivastava, A.

Microwave-Assisted Synthesis of Boron and Nitrogen co-doped Reduced Graphene Oxide for the Protection of Electromagnetic Radiation in Ku-Band. *ACS Appl. Mater. Interfaces* **2015**, *7*, 19831–19842.

3. Yeom, D.Y.; Jeon, W.; Tu, N.D.K.; Yeo, S.Y.; Lee, S.S.; Sung, B.J.; Chang, H.; Lim, J.A.; Kim, H. High-concentration boron doping of graphene nanoplatelets by simple thermal annealing and their supercapacitive properties. *Sci. Rep.* **2015**, *5*.
